# Supplementary material for: Learning curve and surgical outcome of robotic assisted colorectal surgery with ERAS program
Source: Sci Rep. 2022 Nov 29;12:20566. doi: 10.1038/s41598-022-24665-w (PMC9709162; doi:10.1038/s41598-022-24665-w)
Supplement: Supplementary file 2 — Supplementary Information 2. [file 41598_2022_24665_MOESM2_ESM.zip › RAL Raw data-Ñ[▒K/099/OP N_ñw╝╨░O▒Kñσ.pdf]

W72 / W72

臺中榮民總醫院
手術室護理記錄

[redacted]

基本資料

- 室別： 開刀房-17室，手術科別： CRS，手術類別： 常規，口期： 2021/07/17
- 進入時間： 0750 ； 麻醉開始時間： 0810 ； Time Out時間： 0925 ； 劃刀時間： 0926 ； 手術結束時間： 1320 送出時間： 1340 ；
- 麻醉方式： GE

  診斷： Malignant tumor of rectum

  手術名稱： Robot Assisted Low Anterior Resection

  手術局部用藥： NIL

  術前預防性抗生素：
  - 抗生素名稱一： Veterin 1GM IVD由麻醉護理師執行
  - 時間： 0915
  - 上次簽章: 陳映庭

  ，追加預防性抗生素：
- 手術醫師： 蔣鋒帆，林陳彥
- 麻醉醫師： VS 蔡志忠
-
  - 刷手護士A ： 李芩瑜，時間： 0750，刷手護士B ： 陳家瑋，時間： 0750，接替刷手一： 陳映庭，時間： 1130
  - 流動護士A ： 陳映庭，時間： 0750，接替流動一： 陳家瑋，時間： 1100，接替流動二： 李芩瑜，時間： 1200

皮膚狀況

A-V Shunt： 無

術前： 不完整

部位(術前)：

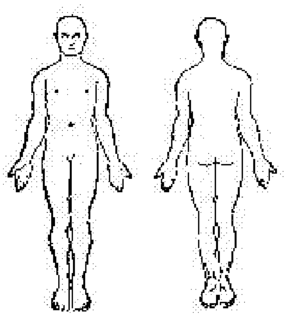

背部約4cm x4cm皮膚紅。

記錄者： 上次簽章: 陳映庭

術後： 不完整

部位(術後)：

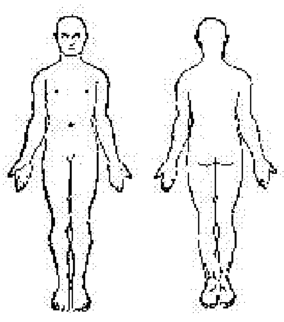

如術前

記錄者： 上次簽章: 李苓瑜

手術準備

手術臥式 單一手術臥式

-
  - 手術臥式： 截石臥式
  - 使用附件： 溫毯 38 ℃，頭圈，腳架
  - 皮膚準備： 10%Aq Betadine，Hibiscrub
  - 電燒： 使用電燒: 電極片位置： 右大腿，雙極電燒，記錄者： 上次簽章: 陳映庭

止血帶T： 無

植入物

- 植入物名稱/號碼/數量/部位： Davinci staper 45-4.3 x2/rectum

, 記錄者： 上次簽章: 李苓瑜

標本送檢

- 細菌培養 無
- 細胞學 無
- 冰凍切片 無
- 病理標本
  組織名稱一： rectum
  ,
  組織名稱二： Distal cut end
  ,
  組織名稱三： IMA root
- 截肢 無

標本交付 標本： 3 袋，標本： 0 盒，標本： 0 瓶，細胞學： 0 管，Culture Tube： 0 管，被交付醫師： CR 林陳彥，

- 記錄者： 李苓瑜 上次簽章: 李苓瑜
- 時間： 1340

傷口術後敷料

傷口敷料： 傷口敷料： 無

- 請於圖上標示部位：

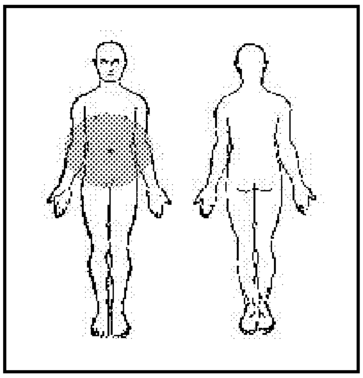

- 傷口敷料： 軟性敷料

導管

導管	天數	導管資訊	插入日期	插入時間	更換日期	拔除日期	拔除時間	拔除原因	預計拔除/更換	預計拔除/更換日期
Foley(1)	1	號碼:16 Fr.; 部位:膀胱;	2021/07/17	0840						
A-line (1)	1	號碼:20 G; 部位:左手;	2021/07/17	1820						
Double Lumen CVC(1)	1	號碼:8 Fr.; 深度:15cm; 部位:jugular (右); 象限:RU;	2021/07/17	0830						

用物計數

- 手術中出血量： Minimal c.c.
- 手術中尿量： 650 c.c.
-
    - 其它： 無

用物計數： 多切口同時縫合

- 1.器械： 正確，2.縫針： 正確，3.刀片： 正確，4.紗布： 正確，5.腹部墊： 未使用，6.紗球： 未使用，7.PEANUTS ： 未使用，8.紗條： 未使用，9.絲帶： 正確，10.針頭： 未使用，11.Vessel loop： 未使用，12.Boots： 未使用，13.Jelco： 未使用，14.棉條： 未使用，
    - 第一次：
        - 刷手護士 ： 陳映庭
        - 流動護士： 上次簽章: 李苓瑜
        - 時間： 1235

    ,
    - 第二次：
        - 刷手護士 ： 陳映庭
        - 流動護士： 上次簽章: 李苓瑜
        - 時間： 1245

    ,
    - 第三次：
        - 刷手護士 ： 陳映庭
        - 流動護士： 上次簽章: 李苓瑜
        - 時間： 1255

備註

備註： Davinci 30度 scope NO.1

- 記錄者： 上次簽章: 陳映庭
- 時間： 1100

填寫護理人員_李苓瑜_

正式摘要

W72 / W72

doctorRank:B
